# Quantifying the effect of IMPEDE-FX packing rate and volume on pressure-normalized principal wall strain in an idealized 3D-printed aneurysm model

**DOI:** 10.1016/j.jvssci.2025.100287

**Published:** 2025-04-15

**Authors:** Baqir Kedwai, Joshua Geiger, Sam Najjar, Joel Kruger, Michael Richards, Chung Yeh, Mary Dennehy, Michael Stoner, Doran Mix

**Affiliations:** a Division of Vascular Surgery, University of Rochester Medical Center, Rochester, NY; b Department of Biomedical Engineering, Rochester Institute of Technology, Rochester, NY; c Shape Memory Medical Inc, Santa Clara, CA

**Keywords:** Aneurysm, Endograft, Embolization plug, Ultrasound, Wall strain

## Abstract

**Background:**

This study aimed to quantify the nonbiologic effects of Shape Memory IMPEDE-FX embolization plug deployment rate and packing volume on pressure-normalized wall strain ($\bar{\varepsilon_{\rho +}}$/PP) of an idealized 3D-printed abdominal aortic aneurysm model.

**Methods:**

An endograft was deployed into an abdominal aortic aneurysm model and connected to an industry-validated hemodynamic simulator. Plugs were deployed into the excluded sac to packing volumes of 100%, 200%, 300%, and 400% under two conditions: (1) sequential and (2) immediate deployment. Axial ultrasound images were taken for each packing volume. Frame-to-frame displacements of the aneurysm wall were measured with ultrasound elastography over one cardiac cycle and normalized to the circuit's pulse pressure to calculate the mean principal strain ($\bar{\varepsilon_{\rho +}}$/PP).

**Results:**

In the 100% packing condition, $\bar{\varepsilon_{\rho +}}$/PP was +113% above baseline at 15 minutes. After sequential deployment to 400%, the $\bar{\varepsilon_{\rho +}}$/PP trended down to +43% above baseline. Immediate packing was associated with a greater $\bar{\varepsilon_{\rho +}}$/PP reduction than sequential packing. When packed immediately to 400%, the $\bar{\varepsilon_{\rho +}}$/PP was −6.7% below baseline.

**Conclusions:**

These modeling data suggest that an immediate deployment strategy and higher plug packing volumes are associated with lower $\bar{\varepsilon_{\rho +}}$/PP, which has been associated with decreased sac growth rates.

**Clinical Relevance:**

The present findings suggest that rapid, high-volume filling of IMPEDE-FX embolization plugs results in a reduction in wall $\bar{\varepsilon_{\rho +}}$/PP, independent of thrombus formation. Fully expanded embolization plugs in aggregate limit pulsatile aortic sac displacement likely contribute to a greater reduction in overall wall strain compared with low packing volumes. These findings may inform clinical application for this device, supporting a rapid and high-volume deployment strategy for greater reduction in $\bar{\varepsilon_{\rho +}}$/PP status post endovascular aneurysm repair.

Endovascular aneurysm repair (EVAR) is the predominant treatment strategy for infrarenal abdominal aortic aneurysms (AAA) in the United States.^1^^,^^2^ EVAR is associated with improved perioperative mortality compared with open surgical repair, but patients require more rigorous long-term monitoring for procedural failure.^3^, ^4^, ^5^, ^6^ Sac regression is a clinical indicator for aortic remodeling after EVAR that has emerged as a strong predictor for long-term survival.^7^

There is no consensus on AAA sac management with persistent growth or endoleak. Many techniques and technologies have been reported to varying degrees of success. Selective management of endoleaks has included strategies such as selective coil embolization of endoleaks and the development of liquid embolization agents such as Onyx.^8^^,^^9^ The development of the Nellix EndoVascular Aneurysm Sealing System (Endologix, Irvine, CA) was designed to prevent endoleaks via polymer-filled bags that filled the entire aneurysm sac.^10^ Unfortunately, a high incidence of graft failure, reintervention, and aneurysm rupture led to the removal of this device from the market.^11^

The relationship between aneurysm rupture and aortic wall biomechanics is well-established. Pulse pressure-normalized maximum mean principal wall strain ($\bar{\varepsilon_{\rho +}}$/PP) is a corollary for aneurysm stability, as multiple studies have shown that stiff, low-strain aneurysms experience lower rates of growth and rupture.^12^, ^13^, ^14^, ^15^ Zottola et al^2^ showed that the $\bar{\varepsilon_{\rho +}}$/PP decreases immediately after EVAR deployment. Interestingly, the authors noted that patients with postoperative type II endoleaks had a greater associated increase in $\bar{\varepsilon_{\rho +}}$/PP after 30 days than those without endoleaks, although the study was not sufficiently powered to detect a statistically significant difference between groups.

The IMPEDE-FX Embolization plug (Shape Memory Medical Inc., Santa Clara, CA) is a self-expanding shape memory polymer (SMP). The plug self-expands to a volume of 1.25 cm^3^, providing space filling with minimal radial force.^16^ The porous structure of this SMP has been shown to enhance clot formation and collagen deposition in animal models.^17^ Current indications for the use of the IMPEDE-FX plug in the United States are limited to peripheral vascular embolization procedures, but there is investigation into its use immediately after EVAR to promote aneurysm sac regression. In human feasibility studies, these plugs have shown significant reduction in sac size and volume when used for preventive sac embolization.^18^^,^^19^ These studies describe a parallel-wire technique to maintain sac access after EVAR, advancing an angled delivery catheter over the jailed wire, and delivering the plugs in a controlled fashion to target branch vessel ostia.^18^ Coupled with the IMPEDE-FX RapidFill system, five plugs can be deployed in rapid succession.

Whereas existing embolization techniques such as coiling function through selective occlusion of branch vessels to treat identified endoleaks, the IMPEDE-FX device is unique in the high-embolic volume provided by each plug. The high-volume space filling of these plugs may function to modulate the biomechanical stress exerted on the aorta when in contact with the aortic wall, though the exact nature of this interaction has not been studied. Mix et al^20^^,^^21^ have developed and validated a technique to assess $\bar{\varepsilon_{\rho +}}$/PP using noninvasive ultrasound elastography (USE). The objective of the present study was to quantify the nonbiologic effects of the IMPEDE-FX packing volume and deployment rate on $\bar{\varepsilon_{\rho +}}$/PP in an idealized 3D-printed silicone aneurysm model using USE. The hypothesis was that a higher packing volume would result in a greater reduction in AAA $\bar{\varepsilon_{\rho +}}$/PP from the plugs working like a dampener, as would a faster deployment rate, from improved distribution of the plugs.

## Methods

### Graft and model preparation

A 3D-printed AAA model was designed as an axisymmetric 28-mm cylinder with a 50-mm spherical aneurysm in the center and uniformly 2.5-mm-thick wall using MeshMixer v3.5 opensource Autodesk (San Rafael, CA) software. BDC Laboratories manufactured this design using an echogenic silicone AAA model, which was validated to have a 5% to 7% compliance/100 mm Hg at 1.2 Hz (Englewood, CO). A 34-mm diameter GORE TAG thoracic stent graft (W. L. Gore & Associates, Newark, DE) was degassed in a negative pressure chamber for 72 hours to improve ultrasound imaging quality. After degassing, the endograft was systematically constrained with silk ties and deployed into the AAA model. This graft model construct was connected to a pulsatile flow circuit. The volume of the aneurysm was calculated to be 65.4 cm^3^ given its design as a perfect sphere with a 50-mm diameter. The volume of the 34-mm stent graft was calculated to be 45.4 cm^3^ across the spherical aneurysm. The volume of the excluded aneurysm sac was, therefore, calculated to be approximately 20.0 cm^3^.

### Flow circuit apparatus

**Fig 1:**
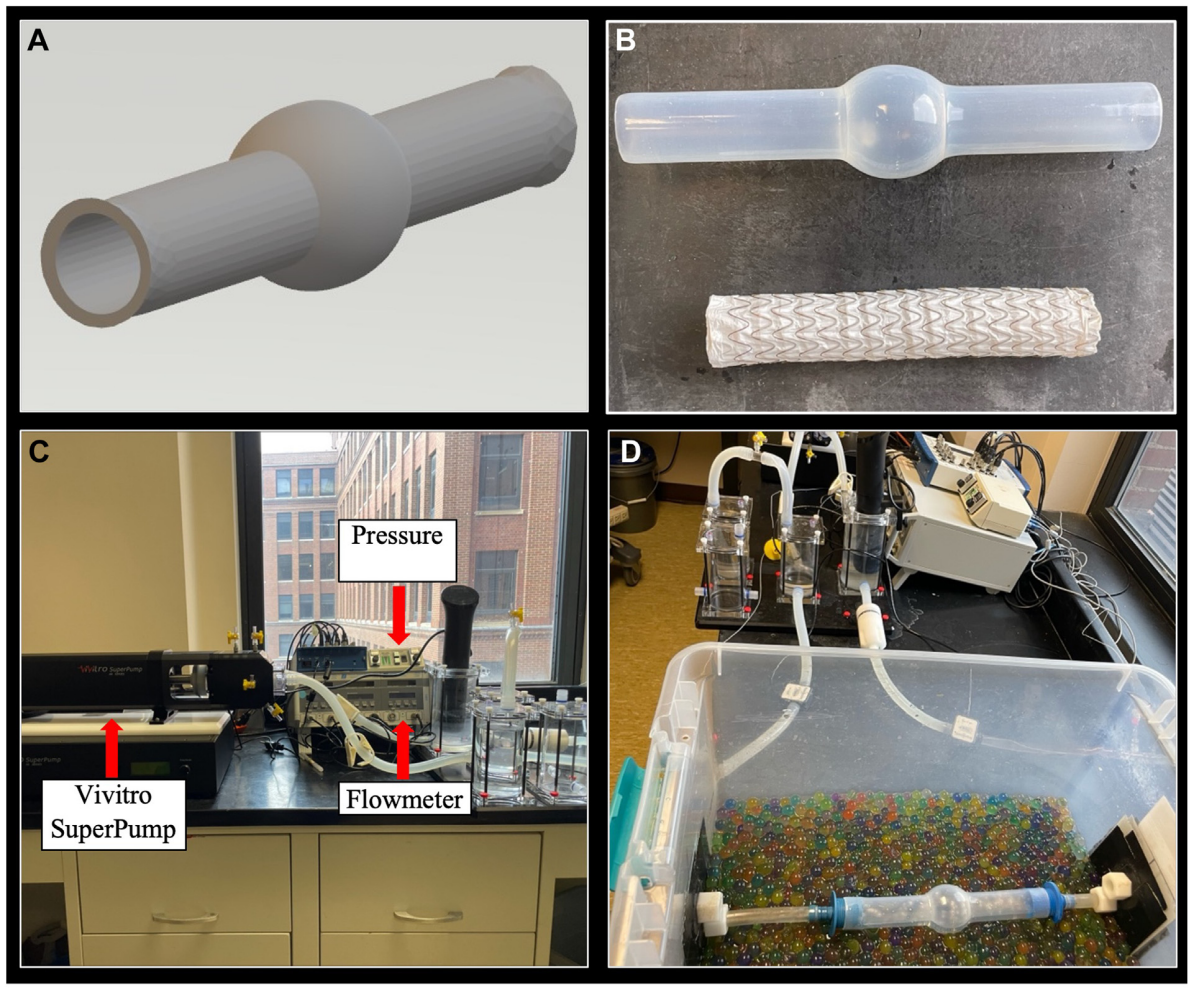
**(A)** Stl. file of idealized abdominal aortic aneurysm (AAA) model. **(B)** A 3D-printed model. **(C)** Flow circuit. **(D)** Model attached to flow circuit.

A detailed description of the authors' flow circuit apparatus has been provided in prior work.^22^ A Vivitro SuperPump (Victoria, BC, Canada) cardiac simulator was connected to both ends of a 30-gallon plastic basin using plastic tubing. The graft model construct was placed into the basin and securely attached proximally and distally to the plastic tubing with circumferential clamps. The basin was filled with water and gel beads for optimal acoustic conditions. Once the model was connected to the flow circuit, water was pumped through the system at 70 beats per minute with an average flow rate of 3.5 L/min. Transonic ME 13 PXN flow probes (Artisan Technology Group, Ithaca, NY) were placed at the inflow and outflow of the model, and the flow rate was continuously monitored by the Transonic TS410 flow meter. Mikro-Cath pressure catheters (Millar Inc, Houston, TX) were placed inside the aortic lumen and excluded aneurysm sac, respectively, to monitor pressures throughout the study. An external heater maintained the fluid's temperature at 37.8°C. The model design and circuit apparatus are depicted in Fig 1.

### Study design

To investigate the effects of plug deployment rate and packing volume on AAA $\bar{\varepsilon_{\rho +}}$/PP, two separate experimental conditions were tested. Packing volumes were calculated based on the excluded aneurysm sac volume of 20.0 cm^3^ and a presumed total expansion volume of the embolization plugs of 1.25 cm^3^. In the first condition, IMPEDE-FX embolization plugs were deployed sequentially to packing volumes of approximately 100% (15 plugs), 200% (30 plugs), 300% (45 plugs), and 400% (60 plugs). In the second condition, embolization plugs were deployed immediately to each packing volume (100%, 200%, 300%, and 400%). Based on prior manufacture testing, each embolization plug was assumed to achieve total expansion within 15 minutes. For this reason, transverse B-mode cine loops were recorded at 5, 10, and 15 minutes after each round of plug deployment. These images were captured at a frequency of 5 MHz using the Ultrasonix Sonix-Touch US System and C7-3/50 convex transducer (Analogic Corporation, Peabody, MA). The ultrasound probe was held at the point of maximal aneurysmal diameter. After each test condition, the model was disconnected from the flow circuit, the graft was carefully extracted using forceps, and the deployed IMPEDE-FX plugs were discarded. The graft was then redeployed into the model with the aid of silk suture constraints.

### Image processing and strain analysis

**Fig 2:**
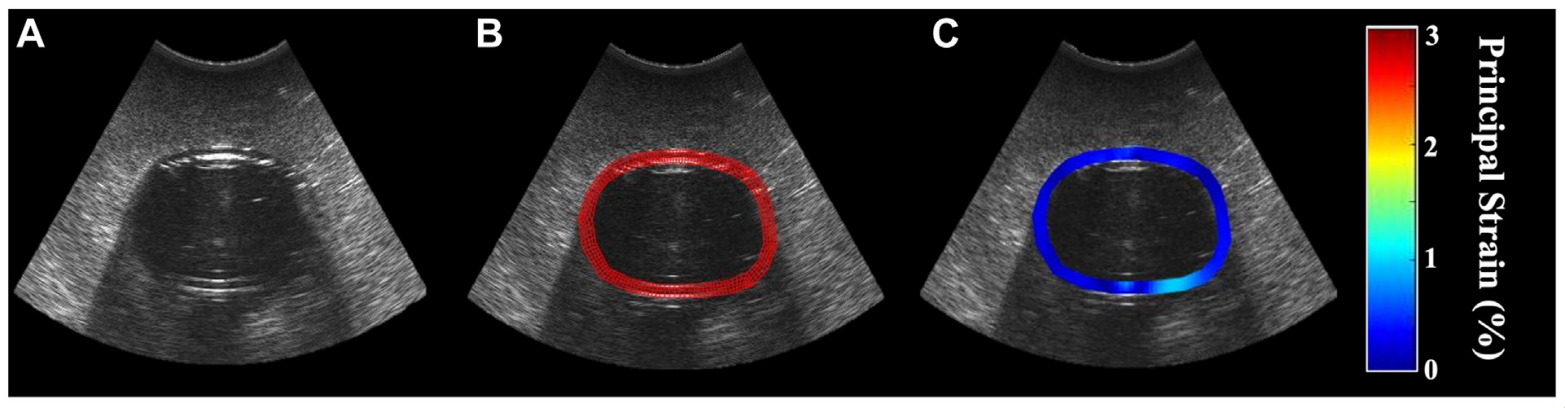
Ultrasound elastography (USE) algorithm, demonstrating **(A)** axial ultrasound image, **(B)** finite element mesh overlay, and **(C)** parametric visualization of wall strain.

Each cine loop was visually inspected to identify one cardiac cycle from end-diastole to end-diastole. The images were then processed and analyzed using the authors' USE technique, which has been previously validated in translational and clinical studies.^20^^,^^21^ First, the inner and outer wall of the AAA were manually selected to delineate the region of interest. A custom MATLAB algorithm was applied to overlay a finite element mesh over the region of interest. The algorithm measured the cumulative frame-to-frame displacement of each element to calculate the mean principal strain ($\bar{\varepsilon_{\rho }}$) at each frame, and the maximum mean principal strain ($\bar{\varepsilon_{\rho +}}$) during a given cardiac cycle. This latter value was then normalized to the circuit's pulse pressure to calculate the pulse pressure-normalized mean principal strain ($\bar{\varepsilon_{\rho +}}$/PP). Fig 2 depicts representative images of each step of the image processing workflow.

## Results

The hemodynamic circuit was operated at a flow rate of 3.5 L/min, a stroke volume of 47.7 mL/pulse, and a temperature of 37.8°C. The mean pulse pressure was 83.4 mm Hg within the AAA lumen and 78.3 mm Hg in the excluded sac. The pulse pressures measured at 5, 10, and 15 minutes for each test condition are summarized in the Table.

**Table:** Pulse pressures measured in the endograft lumen and excluded aneurysm sac at 5-, 10-, and 15-minute time intervals for each deployment condition and packing volume

Condition	Packing volume, %	Time, minutes	Lumen pulse pressure, mm Hg	Sac pulse pressure, mm Hg
Stepwise deployment	100	5	82.2	78.1
		10	82.0	77.6
		15	83.3	78.3
	200	5	83.1	79.1
		10	82.7	78.7
		15	83.0	78.8
	300	5	82.8	79.0
		10	83.4	79.0
		15	83.0	78.8
	400	5	85.3	79.4
		10	83.7	79.0
		15	84.9	80.0
Immediate deployment	100	5	82.2	78.1
		10	82.0	77.6
		15	83.3	78.3
	200	5	78.5	71.8
		10	78.1	71.7
		15	78.7	71.8
	300	5	92.3	85.1
		10	86.7	84.3
		15	92.4	85.2
	400	5	83.4	76.7
		10	82.0	76.3
		15	82.7	77.1

**Fig 3:**
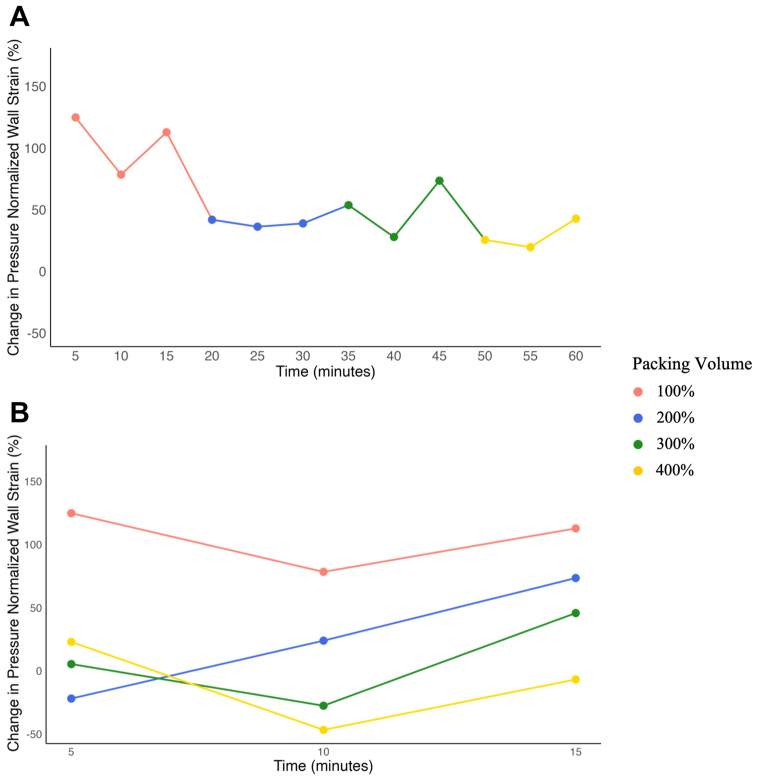
Change in pressure-normalized wall strain over time at multiple plug packing volumes in the **(A)** stepwise and **(B)** immediate deployment conditions.

In the first experimental condition, plugs were deployed sequentially to packing volumes of 100%, 200%, 300%, and 400%. At 100% packing, the $\bar{\varepsilon_{\rho +}}$/PP was +124% above baseline at 5 minutes before normalizing to +113% at 15 minutes. As plugs were deployed to increasing packing volumes, $\bar{\varepsilon_{\rho +}}$/PP trended toward baseline. These findings are summarized in Fig 3, *A*. In the second condition, plugs were deployed immediately to each packing volume (100%, 200%, 300%, and 400%). As the packing volume increased, the $\bar{\varepsilon_{\rho +}}$/PP measured at 15 minutes decreased. At 200% packing volume, the $\bar{\varepsilon_{\rho +}}$/PP was +73% above baseline. At the 300% and 400% packing volume, the 15-minute $\bar{\varepsilon_{\rho +}}$/PP was +45% and −6.7% from baseline, respectively. The results of condition 2 are illustrated in Fig 3, *B*.

## Discussion

The complex interplay between aortic wall biomechanics and AAA sac behavior is an emerging area of research in vascular surgery. Although the AAA wall is typically stiffer than healthy aorta and, therefore, subject to lower strain, compliant AAAs are more likely to rupture. Wilson et al previously showed that a 10% decrease in aortic wall elastic modulus was associated with a nearly 30% increase in rupture risk.^17^ Meanwhile, Zottola et al^23^ reported that intermediate $\bar{\varepsilon_{\rho +}}$/PP AAAs grow more rapidly than low and high-strain aneurysms. Similarly, sac behavior status post EVAR is likely mediated through mechanical changes on the wall in addition to the known biological remodeling. Consistent with prior literature, our group has shown a significant decrease in AAA $\bar{\varepsilon_{\rho +}}$/PP after endograft implantation.^2^ In patients with persistent sac growth requiring secondary intervention such as endoleak embolization, evaluating aortic $\bar{\varepsilon_{\rho +}}$/PP may provide insight into the long-term success or failure of these procedures and the likelihood of sac regression.

Our findings suggest that high-volume space filling of IMPEDE-FX plugs, at a packing density of 400%, can reduce aneurysm sac $\bar{\varepsilon_{\rho +}}$/PP. We hypothesize that the radial force exerted by the embolization plugs acts as a strain dampener by limiting sac displacement experienced with each aortic pulsation. As the plugs are packed at greater densities, the radial force of the plugs in aggregate more effectively reduces the pulsatile strain experienced on the aneurysm wall. This proposed mechanism may also explain the initial increase in baseline $\bar{\varepsilon_{\rho +}}$/PP in both test conditions. At low packing volumes, the dead space of the aneurysm sac is too large for the SMPs to act in aggregate. Thus, the intrasac volume increases with the introduction of each plug, resulting in an overall increase in $\bar{\varepsilon_{\rho +}}$/PP exerted on the aortic wall, without the strain-dampening effect of the fully expanded plugs in aggregate.

With respect to the deployment rate, we hypothesize that the immediate deployment strategy is associated with a greater reduction in $\bar{\varepsilon_{\rho +}}$/PP owing to more optimal plug distribution. Stepwise deployment may bias the displacement of subsequent plugs, resulting in uneven distribution. Conversely, immediate deployment allows for the accumulation of the plugs into high-volume regions of the aortic sac.

The present findings may inform the optimal clinical application of these devices. Most evidently, the present data support the use of very high packing volumes, up to 400% greater than the volume of the sac, to obtain a net reduction in sac wall strain as well as rapid deployment of the plugs to allow for more effective filling of high-volume regions of the sac. At the same time, it is important to consider potential trade-offs to pursuing a high packing volume strategy. At a certain threshold, the increased intra-sac pressure from overpacking may negate the strain-dampening effects of the embolization pugs, increasing the stress exerted on the excluded aneurysm sac and potentially leading to sac rupture. Furthermore, overpacking may contribute to endograft instability and the subsequent development of type I endoleaks.

### Limitations

This modeling study allowed the authors to examine the mechanical interactions of the IMPEDE-FX plug in isolation without confounding thrombus formation. However, studying the interaction between IMPEDE-FX plugs, thrombus, and wall strain would provide valuable insight into the true biomechanical impact of this device on the aneurysm wall. It has been posited that intraluminal thrombus acts to diminish the strain exerted on the aortic wall.^24^ Preclinical data on the IMPEDE-FX plugs have demonstrated that the device enhances thrombus formation, with the scaffold-like structure of the plugs facilitating clotting and collagen formation.^17^ The authors' hypothesize that the plugs may have a synergistic effect on reducing wall strain in the presence of intra-luminal thrombus by increasing clot formation and potentiating remodeling.

The pulsatile flow simulator is inherently limited in its ability to replicate the dynamic complexities of the human cardiovascular system. Additionally, the idealized axisymmetric model is not representative of the varied and complex anatomy of degenerative aortic aneurysms, which limits the in vivo generalizability of our findings. Another notable limitation of the present work is the absence of statistical analyses to study the variability of the results. Each condition was tested only once due to the limited availability of IMPEDE-FX plugs, which precluded the evaluation of the data's dispersion. The study is, therefore, primarily descriptive in nature and its findings cannot be extrapolated further. Overall, the present findings provide a preliminary insight into the biomechanical interactions between the IMPEDE-FX plug and vessel wall, and may inform future clinical applications for the novel device.

## Conclusions

This study examined the effects of IMPEDE-FX embolization plug packing rate and volume on aortic $\bar{\varepsilon_{\rho +}}$/PP in an idealized AAA model. High packing volumes and immediate deployment of IMPEDE-FX plugs result in greater aortic $\bar{\varepsilon_{\rho +}}$/PP reduction, suggesting that these self-expanding plugs confer a strain-dampening effect on the aneurysm sac. Clinical application of these principles may enhance aneurysm sac regression and contribute to improved outcomes after IMPEDE-FX embolization.

## Funding

The 10.13039/100000002National Institutes of Health supported this work through the following grants: KL2 TR001999, R21 EB018432, and TL1 TR002000.

## Disclosures

Shape Memory Medical Inc. donated the embolization plug devices for this study. C.Y. and M.D. are employed by Shape Memory Medical Inc, and were involved in the interpretation of the present findings, as well as critical revisions but were not involved in the design, data collection or analysis of the study.
